# Exposure to Elevated Nitrogen Dioxide Concentrations and Cardiac Remodeling in Patients With Dilated Cardiomyopathy

**DOI:** 10.1016/j.cardfail.2021.11.023

**Published:** 2022-06

**Authors:** DANIELA Fecht, MARC CHADEAU-HYAM, RUTH OWEN, JOHN GREGSON, BRIAN P. HALLIDAY, Amrit S. Lota, JOHN GULLIVER, JAMES S. WARE, DUDLEY J. PENNELL, FRANK J. KELLY, ANOOP S.V. SHAH, MARK R. MILLER, DAVID E. NEWBY, SANJAY K. PRASAD, UPASANA TAYAL

**Affiliations:** 1 MRC Centre for Environment and Health, School of Public Health, Imperial College London, London, UK; 2 Department of Medical Statistics, London School of Hygiene and Tropical Medicine, London, UK; 3 Royal Brompton Hospital and Harefield NHS Trust, London, UK; 4 National Heart Lung Institute, Imperial College London, London, UK; 5 MRC London Institute of Medical Sciences, Imperial College London, London, UK; 6 Centre for Environmental Health and Sustainability & School of Geography, Geology and the Environment, University of Leicester, Leicester, UK; 7 NIHR Health Protection Unit in Environmental Exposures and Health, Imperial College London, London, UK; 8 Department of Non-communicable Disease, London School of Hygiene & Tropical Medicine, London, UK; 9 BHF Centre for Cardiovascular Science, University of Edinburgh, Edinburgh, UK

**Keywords:** Nitrogen dioxide, particulate matter, heart, cardiomyopathy, heart failure

## Abstract

**Background:**

Empirical evidence suggests a strong link between exposure to air pollution and heart failure incidence, hospitalizations, and mortality, but the biological basis of this remains unclear. We sought to determine the relationship between differential air pollution levels and changes in cardiac structure and function in patients with dilated cardiomyopathy.

**Methods and Results:**

We undertook a prospective longitudinal observational cohort study of patients in England with dilated cardiomyopathy (enrollment 2009–2015, *n* = 716, 66% male, 85% Caucasian) and conducted cross sectional analysis at the time of study enrollment. Annual average air pollution exposure estimates for nitrogen dioxide (NO_2_) and particulate matter with diameter of 2.5 µm or less (PM_2.5_) at enrolment were assigned to each residential postcode (on average 12 households). The relationship between air pollution and cardiac morphology was assessed using linear regression modelling. Greater ambient exposure to NO_2_ was associated with higher indexed left ventricular (LV) mass (4.3 g/m^2^ increase per interquartile range increase in NO_2_, 95% confidence interval 1.9–7.0 g/m^2^) and lower LV ejection fraction (–1.5% decrease per interquartile range increase in NO_2_, 95% confidence interval –2.7% to –0.2%), independent of age, sex, socioeconomic status, and clinical covariates. The associations were robust to adjustment for smoking status and geographical clustering by postcode area. The effect of air pollution on LV mass was greatest in women. These effects were specific to NO_2_ exposure.

**Conclusions:**

Exposure to air pollution is associated with raised LV mass and lower LV ejection fraction, with the strongest effect in women. Although epidemiological associations between air pollution and heart failure have been established and supported by preclinical studies, our findings provide novel empirical evidence of cardiac remodeling and exposure to air pollution with important clinical and public health implications.

## Highlights

• Nitrogen dioxide air pollution is associated with adverse cardiac remodeling in patients with dilated cardiomyopathy.
• Women seem to be more susceptible to these effects.
• Patients with dilated cardiomyopathy should be aware of the link between air pollution and their cardiovascular health so that they may take steps to minimize their exposure to pollutants.

## Graphical abstract

**Figure:**
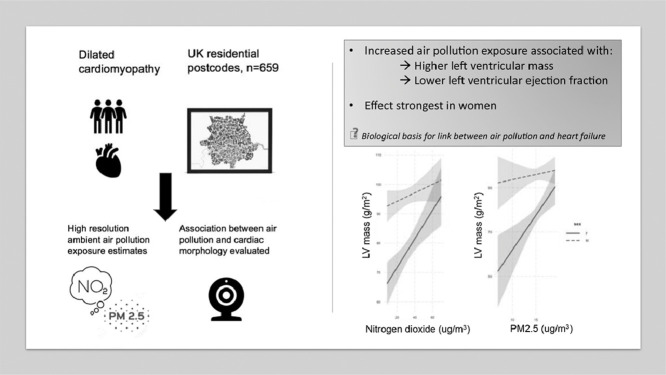

Air pollution is a major cause of premature mortality and is the leading global cause of death from noncommunicable disease after smoking. The World Health Organisation estimates that 31% of cardiovascular disease is attributable to environmental factors, of which air pollution is the most important.^1^

Heart failure (HF) affects 26 million people worldwide and is a major morbidity and mortality burden^2^. Empirical evidence suggests that exposure to ambient air pollution, in particular NO_2_ and particulate matter with aerodynamic diameter of less than 2.5 µm (PM_2.5_), is strongly associated with HF incidence, hospitalization, and mortality,^3^^,^^4^ as well as mortality after heart transplantation.^5^ However, HF is a highly heterogeneous condition. The individual characterization of participants in most epidemiological studies has been limited. As such, the biological basis of the exposure–outcome relationship between air pollution and adverse HF effects remains speculative. One potential mechanism which has been demonstrated in individuals without cardiac disease is adverse ventricular remodelling.^6^, ^7^, ^8^, ^9^

The second most common cause of HF after coronary artery disease is dilated cardiomyopathy (DCM), affecting up to 1 in 250 individuals.^10^ Although there is evidence of air pollution exacerbating coronary artery disease, there is no current evidence for an association between air pollution and DCM, a key contributor to HF. DCM is characterized by LV dilatation and impairment of cardiac function, in the absence of coronary artery disease or abnormal loading conditions. DCM can have heterogenous manifestations, particularly in disease severity, even among individuals who carry the same genetic variant, suggesting that there are environmental modifiers of the disease.

In this study, we leveraged precision phenotyping to evaluate the biological basis of the robust epidemiological and preclinical links between air pollution and HF. We assessed whether higher exposure to ambient air pollution is associated with adverse cardiac remodeling through detailed phenotyping in a large cohort of patients with DCM.

## Methods

### Study Population

Participants comprised 716 patients with a clinical diagnosis of DCM confirmed by gadolinium-enhanced cardiovascular magnetic resonance (CMR) prospectively enrolled in the National Institute for Health Research Royal Brompton Hospital Cardiovascular Biobank project between 2009 and 2015.^11^ This is one of the largest and most comprehensively phenotyped cohorts of patients with DCM. Crucially, the precise phenotyping enabled the generation of a truly nonischemic cohort of patients, which allows an evaluation of the effects of air pollution and heart structure without the confounding variable of coronary artery disease. Patients were recruited via a broad network of more than 30 referring hospitals across London and the south of England. Patients were recruited as close as possible to the time of DCM diagnosis (median interval between DCM diagnosis and baseline study CMR scan which defined enrollment was 0.1 years, interquartile range 0.0–0.6 years).

All patients provided written informed consent. The study was approved by the regional ethics committee.

### Outcome Characterization

The diagnosis of DCM was made based on CMR evidence of LV dilation and systolic impairment with reference to age, sex, and body surface area adjusted nomograms.^12^ Exclusion criteria for DCM included a history of uncontrolled systemic hypertension, coronary artery disease (>50% stenosis in ≥1 major epicardial arteries or previous percutaneous coronary intervention or coronary artery bypass grafting), chronic excess alcohol consumption meeting criteria for alcoholic cardiomyopathy (>80 g/day for >5 years^13^), systemic disease known to cause DCM, pericardial disease, congenital heart disease, infiltrative disorders (eg, sarcoidosis), recent acute presentation of myocarditis, or significant primary valvular disease.^14^^,^^15^

The analysis was restricted to patients with UK residential postcodes. All patients underwent clinical screening at recruitment to the study and CMR assessment of cardiac volumes, function and fibrosis (1.5T, Siemens Sonata or Avanto scanners, Siemens Medical Systems, Erlangen, Germany) as described previously.^11^ All CMR data were analyzed using a standardized methodology and analysis package by operators blinded to pollution data. Patients also had targeted cardiac genetic analysis (TruSight Cardio Sequencing kit, Illumina, San Diego, CA). Truncating variants in the titin gene are the most common genetic variant in DCM and were curated as previously described.^16^

Socioeconomic status was assessed for each participant based on their place of residence using the English Index of Multiple Deprivation 2015 (IMD2015), the official measure of deprivation for small areas (a small area is a standard statistical geography designed to be of a similar population size, approximately 1500 residents). The IMD2015 is a weighted average of 7 deprivation domains: income deprivation, employment deprivation, health deprivation and disability, education skills and training deprivation, barriers to housing and services, living environment deprivation, and crime. Information on smoking status was collected at enrolment and defined as current, ex-smoker, or never smoker.

### Air Pollution Exposure

We assigned long-term air pollution exposure based on previously modelled annual ambient air pollution concentrations for Great Britain using land use regression models. Models are described in detail elsewhere.^17^^,^^18^ In brief, models combined air pollution measurements with land use and traffic variables to predict NO_2_ concentrations for 2009 at 200 m resolution^17^ and PM_2.5_ for 2010 at 100 m resolution.^18^ Both models were extensively validated against monitoring data and showed good agreement between modelled and measured air pollution levels.^17^^,^^18^ We adopted a method for forward extrapolation of 2009–2010 air pollution exposure to the time of the study CMR scan using information from the national air pollution monitoring network (Supplementary Materials).^17^ We assigned air pollution estimates to participant's residential postcode centroid which represent on average 12 households, enabling high-resolution location mapping for each participant. Postcodes were geocoded using year-specific coordinates based on the UK Small Area Health Statistics Unit historical postcode database.

### Statistical Analysis

Continuous data were summarized using median and interquartile range and compared using the Kruskal Wallis test; categorical data as count and percentages and compared using the Fisher exact test.

Pearson's correlation coefficient (r) was used to test the association between the two air pollutants. Univariable linear regression analyses were conducted to examine the associations of each air pollutant (NO_2_ and PM_2.5_) with LV ejection fraction, LV mass, LV end-diastolic and LV end-systolic volume, LV stroke volume, right ventricular (RV) ejection fraction, RV end-diastolic volume, RV end-systolic volume and RV stroke volume. Except for the ejection fraction, all cardiac measurements were indexed to body surface area. Using the conservative Bonferroni correction for multiple testing, the per test significance level for the univariable analyses was 0.006 and ensured a control of the family wise error rate of less than 0.05 for each exposure separately. In multivariable analyses, baseline models were built for LV ejection fraction and LV mass (outlined further in the Supplementary Methods). We adjusted for age, sex, and socioeconomic status, as well as clinical covariates that were associated with LV mass and LV ejection fraction. All available clinical covariates including comorbidities and medication were considered for model building. The final multivariable models only include those variables that were significantly associated with the outcome of interest. Results for linear regression analyses are presented as change in phenotype (LV ejection fraction or LV mass) per interquartile range increase in pollutant with 95% confidence intervals (CI). We additionally performed sex-stratified analyses. When investigating an interaction between biological sex and NO_2_ and PM_2.5_, we included a multiplicative interaction term. Regression model assumptions were assessed using residuals plots. Sensitivity analyses were conducted by (1) restricted cubic spine transformation of exposure variables to investigate nonlinear relationships using the splines package in R and (2) linear mixed effects modelling using the lme4 package in R, with a random intercept for postcode area to assess for clustered observations within a geographical area that may be highly correlated. Postcode area is based on the first 2 alpha characters of the postcode (121 postcode areas in the UK). The intraclass correlation coefficient was calculated by dividing the between group variance by the total variance (sum of between group variance and residual variance).

All statistical analyses were conducted in the R environment, version 3.6.2 (R Foundation for Statistical Computing, Vienna, Austria).

## Results

From the total cohort of 716 prospectively recruited consecutive patients with DCM who were enrolled into the study between 2009 and 2015, 659 could be assigned a geographical location via a UK residential postcode and consequently air pollutant estimates (Table 1). We were not able to geolocate 57 patients owing to their postcode being missing, partially missing, or incorrect.

**Table 1:** Cohort Demographics and Cardiac Imaging Variables at Baseline

	Total Cohort (*N* = 659)
Age (years)	54 [44–64]
Male sex (%)	435 (66)
Caucasian (%)	568 (86)
Controlled hypertension (%)	192 (29)
Diabetes mellitus (%)	80 (12)
Body surface area (m^2^)	2.00 [1.80–2.20]
Resting heart rate (bpm)	73 [63–85]
Systolic blood pressure (mm Hg)	121 [110–134]
Diastolic blood pressure (mm Hg)	73 [65–82]
NYHA functional class	
1	276 (44)
2	255 (40)
3	92 (15)
4	8 (1)
Beta-blocker use	466 (71)
ACE inhibitor use	527 (80)
Aldosterone antagonist use	240 (36)
Diuretic use	304 (46)
Family history of DCM	106 (16)
Family history of sudden cardiac death	98 (15)
Previous history of myocarditis	26 (4)
Any pathogenic/likely pathogenic genetic variant in a gene linked to DCM	93 (14)
Truncating variant in the titin gene	80 (12)
LV ejection fraction (%)	40 [30–49]
LV end-diastolic volume indexed to BSA (mL/m^2^)	117 [102–143]
LV end-systolic volume indexed to BSA (mL/m^2^)	69 [53–97]
LV stroke volume indexed to BSA (mL/m^2^)	48 [38–57]
LV mass indexed to BSA (g/m^2^)	87 [74–106]
RV ejection fraction (%)	54 [44–61]
RV end-diastolic volume indexed to BSA (mL/m^2^)	85 [70–101]
RV end-systolic volume indexed to BSA (mL/m^2^)	40 [29–53]
RV stroke volume indexed to BSA (mL/m^2^)	43 [35–53]
Left atrial volume indexed to BSA (mL/m^2^)	55 [45–71]
Midwall myocardial fibrosis (detected on late gadolinium enhancement imaging)	227 (34)
Maximum LV wall thickness (mm)	10 [8–11]
Mean septal wall thickness (mm)	8 [6–9]
Mean lateral LV wall thickness (mm)	5 [4–6]

Data are shown as median [interquartile range] and counts (percentages). BSA, body surface area. NYHA, New York Heart association functional class, ACE, angiotensin-converting enzyme.

### Air Pollution Exposure

**Fig. 1:**
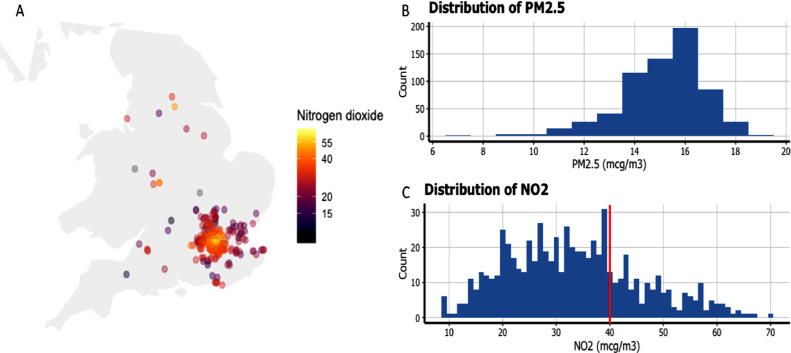
Pollutant exposure in the cohort. (**A**) Location of participants across the UK color coded by ambient exposure to NO_2_ (μg/m^3^) and distribution of **(B**) PM_2.5_ (μg/m^3^) and (**C**) NO_2_ (μg/m^3^) across the cohort. Over one quarter of the cohort had NO_2_ exposure higher than European legal limits (40 μg/m^3^, indicated using the red line).

The median NO_2_ concentration was 32.4 (interquartile range 24.1–40.6) μg/m^3^ and the median PM_2.5_ exposure was 15.4 (interquartile range 14.3–16.3) μg/m^3^. There was a moderate positive correlation between the two pollutants (r = 0.44, *P* < .00001). In total, 169 patients (26%) had an NO_2_ exposure of greater than 40 μg/m^3^, the European Union annual limit (Fig. 1). A comparison of phenotype characteristics between this subgroup and those with NO_2_ exposure within legal limits is shown in Supplementary Table 1.

Baseline characteristics of the study population stratified by exposure to NO_2_ (Table 2) demonstrated that patients were older and more likely to be non-Caucasians in the highest tertile of exposure, but there were no differences in sex between the exposure groups. There was a greater proportion of patients with a history of controlled hypertension in the highest NO_2_ exposure group. Compared to the other groups, patients in the highest exposure group also had higher LV mass. There was no correlation between baseline systolic blood pressure measured on the day of enrolment (mm Hg ) and air pollution (NO_2_: r = 0.008, *P* = .85; PM_2.5_: r = –0.004, *P* = .91) or between baseline systolic blood pressure (mm Hg) and LV mass (r = 0.03, *P* = .40).

**Table 2:** Baseline Cohort Characteristics and Imaging Variables Stratified by Tertiles of Exposure to NO_2_

	High NO_2_ tertile (38.2–69.6 μg/m^3^), *n* = 219	Medium NO_2_ tertile (26.9–38.1 μg/m^3^), *n* = 220	Low NO_2_ tertile (8.6–26.8 μg/m^3^), *n* = 220	*P* Value
Age (years)	57 [45–66]	55 [44–64]	53 [44–62]	.04
Male sex (%)	142 (65)	150 (68)	143 (65)	.71
Caucasian (%)	167 (76)	201 (91)	200 (91)	.008
Hypertension (%)	79 (36)	58 (26)	55 (25)	.02
Diabetes mellitus (%)	21 (10)	31 (14)	28 (13)	.33
Body surface area (m^2^)	2.00 [1.80–2.10]	2.00 [1.80–2.20]	2.00 [1.80–2.12]	.51
Resting heart rate (bpm)	72 [61–86]	74 [64–87]	72 [63–83]	.24
Systolic blood pressure (mm Hg)	125 [110–137]	120 [109–131]	122 [112–134]	.25
Diastolic blood pressure (mm Hg)	73 [65–84]	71 [64–81]	74 [65–82]	.38
Index of multiple deprivation quintile				<.001
1 (most deprived)	24 (14)	7 (4)	15 (8)	
2	47 (27)	25 (13)	36 (19)	
3	35 (20)	39 (21)	33 (17)	
4	37 (21)	58 (31)	46 (24)	
5 (least deprived)	31 (18)	60 (32)	63 (33)	
LV ejection fraction (%)	38 [28–48]	40 [30–50]	44 [31–51]	.05
LV end-diastolic volume indexed to BSA (mL/m^2^)	118 [103–144]	118 [104–141]	116 [101–141]	.45
LV end-systolic volume indexed to BSA (mL/m^2^)	72 [55–100]	70 [53–96]	65 [51–96]	.14
LV stroke volume indexed to BSA (mL/m^2^)	47 [37–56]	48 [37–57]	49 [39–57]	.39
LV mass indexed to BSA (g/m^2^)	89 [77–108]	86 [74–107]	83 [72–100]	.02
RV ejection fraction (%)	54 [43–62]	54 [44–60]	54 [44–61]	.84
RV end-diastolic volume indexed to BSA (mL/m^2^)	85 [69–100]	86 [71–101]	84 [69–102]	.82
RV end-systolic volume indexed to BSA (mL/m^2^)	40 [28–53]	41 [30–52]	40 [28–53]	.78
RV stroke volume indexed to BSA (mL/m^2^)	43 [35–52]	43 [35–53]	44 [36–53]	.89
Left atrial volume indexed to BSA (mL/m^2^)	55 [44–72]	55 [46–70]	56 [46–71]	.91
Mid wall myocardial fibrosis (detected on late gadolinium enhancement imaging)	78 (36)	77 (35)	72 (33)	.8

Data are shown as median [interquartile range] and counts (percentages) and compared using the Kruskal–Wallis test or Fisher's exact test respectively. BSA, body surface area. The range of NO_2_ concentrations in each tertile is shown in brackets.

### Air Pollutants and Cardiac Morphology and Function

#### NO_2_ Exposure and LV Mass

Higher ambient exposure to NO_2_ was associated with higher indexed LV mass (4.5g/m^2^ increase per interquartile range increase in NO_2_, 95% CI = 1.8–7.1 g/m^2^, *P* = .001) in a univariable linear regression analysis.

**Fig. 2:**
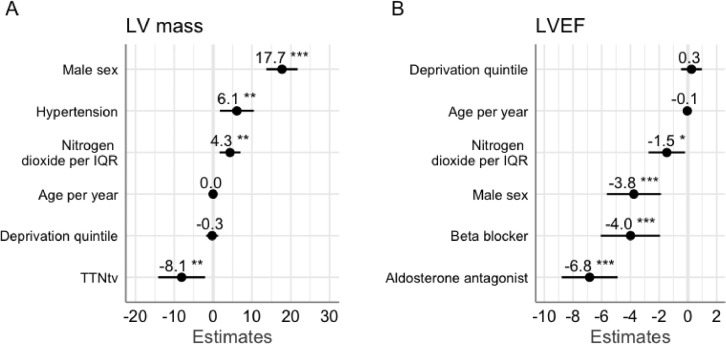
Pollutant exposure and cardiac structure and function. Forest plots of the multivariable linear regression models describing the association of NO_2_ exposure and cardiac structure. (**A**) Increasing exposure to NO_2_ is associated with increased LV mass (**A**) and reduced LV ejection fraction (**B**). TTNtv, truncating variants in the titin gene. Asterisks indicate the significance level of the *P* values. The interquartile range for NO_2_ concentration was 24.1–40.6 μg/m^3^ and for PM_2.5_ was 14.3–16.3 μg/m^3^.

In our cohort, sex, hypertension, and the presence of a truncating variant in the titin gene were associated with LV mass. After adjustment for these covariates, as well as age and socioeconomic status, exposure to higher NO_2_ concentrations was associated with higher LV mass (4.3 g/m^2^ increase per interquartile range increase in NO_2_, 95% CI 1.9–7.0 g/m^2^, *P* = .002) (Fig. 2 and Supplementary Table 4).

There was no evidence of an interaction of exposure to both NO_2_ and PM_2.5_ on LV mass (*P* interaction = .08).

#### NO_2_ Exposure and LV Ejection Fraction

Ambient exposure to higher NO_2_ concentrations was associated with lower LV ejection fraction (–1.9% decrease per interquartile range increase in NO_2_, 95% CI –3.1 to –0.6%, *P* = .004) in a univariable linear regression analysis.

The association between exposure to higher levels of NO_2_ and a reduction in LV ejection fraction was only modestly attenuated after adjustment for the variables (age, sex, socioeconomic status, beta-blocker use, and aldosterone antagonist use) which were associated with LV ejection fraction in this cohort (–1.5% decrease per interquartile range increase in NO_2_, 95% CI –2.7 to –0.2%, *P* = .02) (Fig. 2 and Supplementary Table 5).

#### Air Pollution Exposure and Other Indices of Cardiac Structure and Function

The association between higher ambient exposure to PM_2.5_ and higher LV mass (2.7 g/m^2^ increase per interquartile range increase in PM_2.5_, 95% CI 0.4–5.2 g/m^2^, *P* = .02) was not significant after adjustment for multiple testing (*P* > .006). The relationship between exposure to higher PM_2.5_ concentrations and indexed LV mass did not change in multivariable linear regression analysis (2.8 g/m^2^ increase per interquartile range increase in PM_2.5_, 95% CI 0.6–5.1, *P* = .02) (Supplementary Table 6).

At nominal significance (*P* < .05), there were links between higher NO_2_ and higher LV end-systolic volume and between higher PM_2.5_ and lower RV end-diastolic volume, but these associations were not significant after adjustment for multiple testing (Supplementary Tables 2 and 3). There was no other association between exposure to NO_2_ or PM_2.5_ and other parameters of cardiac structure and function (Supplementary Figs. 1 and 2).

#### Adjustment for Smoking Status

Although smoking status was not found to be associated with indices of cardiac structure or function, it was thought to be a potentially important confounder therefore additional analyses were performed. There were 63 current smokers and 157 ex-smokers in the cohort. In multivariable analysis adjusting for smoking status in addition to the previously outlined variables, exposure to NO_2_ remained associated with increased LV mass (4.0 g/m^2^ increase per interquartile range increase in NO_2_, 95% CI 1.1–6.9 g/m^2^, *P* = .007) and reduced LV function (–1.5% decrease in LV ejection fraction per interquartile range increase in NO_2_, 95% CI –2.9 to –0.2%, *P* = .03). However, after adjusting for smoking status, there was only an apparent trend for increasing exposure to PM_2.5_ to be associated with increasing LV mass (2.9 g/m^2^ increase per interquartile range increase in PM_2.5_, 95% CI –0.2 to 5.9, *P* = .07).

### Evaluating Sex-specific Effects of Pollution Exposure on Cardiac Structure

**Fig. 3:**
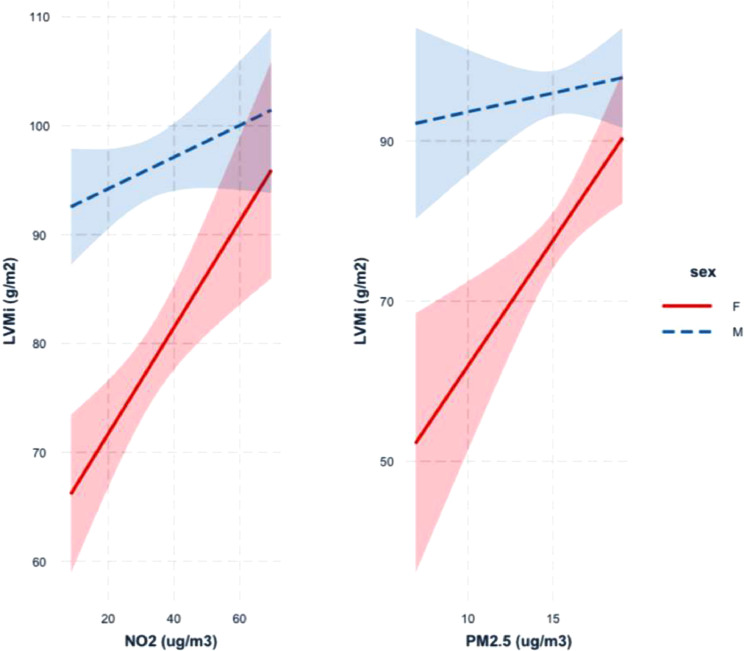
Biological sex as a modifier of the relationship between pollutant exposure and LV mass. The association between increasing pollutant exposure to either NO_2_ (left) or particulate matter (right) and increasing indexed LV mass (LVMi) is stronger for women (red lines) compared with men (dotted blue lines). Interaction plots shown for the multivariable regression model, adjusting for age, socioeconomic status, hypertension and a titin truncating variant, variables that were associated with LV mass.

Although we controlled for sex as a biological variable in the multivariable linear regression analyses, we additionally sought to investigate the potential differences between male and female participants in stratified analyses. There was no difference in age at study enrollment between male and female participants (mean age males 53.1 years, females 53.9 years, *P* = .52). The effect of increasing air pollution on LV mass was greater for women compared to men (Fig. 3). Although men had higher indexed LV mass at baseline (mean LV mass males 94.9 g/m^2^, females 79.4 g/m^2^, *P* < .0001), women seemed to be most vulnerable to the effects of increasing air pollution exposure. For NO_2_ the increase in indexed LV mass was 7.9 (95% CI 3.5–12.3) g/m^2^ per interquartile range increase in women compared with 2.4 g/m^2^ (95% CI –0.8 to 5.7 g/m^2^) in men (*P* interaction = .04). For PM_2.5_ the increase in indexed LV mass in women was 6.1 g/m^2^ (95% CI 2.2–9.9 g/m^2^) per interquartile range increase and 0.7 g/m^2^ (95% CI 2.3–3.7 g/m^2^) in men (*P* interaction = .03). There was no evidence of an interaction of biological sex and exposure to NO_2_ for LV ejection fraction (*P* interaction = .30).

### Sensitivity Analyses

Assumptions of linear models were not violated. In additional analyses, nonlinear concentration-response relationships were explored using cubic spline regression and no alternative concentration-response relationship was observed (Supplementary materials).

To assess for clustered observations within a geographical area that may be highly correlated, we performed mixed effects modelling with postcode area as the grouping variable. The between cluster variation was close to zero for all models, suggesting that a fixed effects model was appropriate and that there was no significant variation between postcode areas (Supplementary Table 7).

## Discussion

This study evaluates the association between cardiac remodeling and exposure to air pollution and provides the first empirical evidence that higher ambient exposure to air pollution is associated with eccentric cardiac hypertrophy in patients with DCM. Harnessing precision phenotyping with CMR, we found that increased exposure to NO_2_ was associated with higher LV mass and lower LV ejection fraction. Women seem to be more susceptible to these adverse effects. Although epidemiological and preclinical studies have shown strong associations between air pollution and HF, this study is the first in humans to provide biological plausibility for these associations. These novel findings contribute to the understanding of DCM disease progression and have clinical implications for the management of patients with HF.

NO_2_ is a gaseous pollutant that mainly originates from vehicle emissions, especially from diesel vehicles, and correlates closely with PM_2.5_ concentrations, which are also primarily generated by road traffic. However, PM_2.5_ can also originate from other local and regional background contributions, including domestic heating and industry. NO_2_ is a surrogate for other air pollutants, such as ultrafine particles or PM_0.1_, which are generated by traffic and contribute to PM_2.5_ concentrations but are currently not routinely measured in the UK. Most adverse cardiovascular health effects from air pollution have been linked to particulate matter although in our cohort we saw the strongest biological associations with NO_2_. Pollution levels in urban areas such as London often exceed the European Union NO_2_ annual limit values and exceedances in daily NO_2_ limits are not uncommon. More than one-quarter of our cohort were exposed to NO_2_ concentrations higher than European legal limits. This finding might explain the more robust evidence for associations between NO_2_ and cardiac remodeling as compared with PM_2.5_ and underlines the importance of road traffic-generated air pollution.

Although the magnitudes of overall population effects were small, the direction and consistency of effects for both LV mass and ejection fraction are compelling. Our results suggest that air pollution is an environmental modifier of the phenotype of DCM. Crucially, public health policy interventions and personal lifestyle changes could mitigate the effect of this and potentially lead to improved health outcomes for patients with DCM or HF as supported by a recent position paper from the World Heart Federation, American College of Cardiology, American Heart Association, and the European Society of Cardiology.^19^ In line with other studies, we have seen that there are no safe exposure limits and deleterious biological effects can be observed below the current legal limits of annual average exposure of 40 µg/m^3^ for NO_2_ and 25 µg/m^3^ for PM_2.5_.^20^^,^^21^

A major strength of our study is the depth of characterization of a large clinical cohort of patients with DCM, enabling the precise evaluation of cardiac structure and function. This is one of the largest and most comprehensively phenotyped cohorts of patients with DCM. Although NO_2_ and PM_2.5_ have been previously linked to incident HF and cardiovascular disease,^3^^,^^22^, ^23^, ^24^, ^25^ our study is the first to demonstrate the association between pollutant exposure and adverse cardiac structure and function in patients with HF secondary to DCM specifically. This is important as the evaluation of the relationship between air pollution and HF may be confounded by the effect of air pollution on coronary artery disease.

Our findings are supported by a number of studies that have evaluated the association between pollutant exposure and cardiac structure in individuals without known previous cardiac disease including the UK biobank^6^ and the Multi-Ethnic Study of Atherosclerosis (MESA)^8^ cohorts. In these studies, closer proximity to main roads was associated with increased LV mass. However, these studies did not quantify air pollutants in the way we have done, and they can only point to subclinical disease, in contrast with our findings. What we show in our current study for the first time is that the relationship between increased pollutant exposure and increased LV mass exists in patients with DCM. This finding is important because DCM is the second most common cause of HF. Therefore, this study shows a potential mechanistic basis to the link between pollutant exposure and HF, namely, increased LV mass. Other studies from the MESA cohort have shown air pollution to be associated with increased RV mass^7^^,^^9^ and RV dysfunction, which we did not find. Notably, even with CMR, estimating the RV mass is prone to great error owing to the thin walls and trabeculation.

Our findings are also supported by population studies assessing the relationship between air pollution and incident HF. In an English national cohort study, one interquartile range change in either PM_10_ or in NO_2_ (3.0 and 10.7 μg/m^3^, respectively) was each independently associated with a hazard ratio for incident HF of 1.06 (95% CI 1.01–1.11) after adjustment for confounders.^22^ A subsequent meta-analysis identified that both NO_2_ and PM_2.5_ were associated with HF hospitalizations and death.^3^ The relationship between pollutant exposure and incident DCM has not yet been established.

The mechanism by which exposure to air pollution leads to adverse cardiac remodeling is likely multifactorial and may act via pathways such as myocardial ischemia, direct cardiomyocyte toxicity, or important mediators such as hypertension.^26^, ^27^, ^28^ Genetic polymorphisms in genes related to vascular function, inflammation, and oxidative stress have been shown to modify the associations between proximity to major roads and LV mass in participants from the MESA cohort.^29^ Our study demonstrates an association between air pollutant exposure and adverse cardiac phenotypes in DCM, but we cannot infer causation. There remains uncertainty as to biological mechanisms that link the inhalation of particles to toxicological effects on the cardiovascular system.^20^ However, previous murine models with long-term (9 months) exposure to PM_2.5_ (approximately 15 μg/m^3^) demonstrated increases in hypertrophic markers leading to adverse ventricular remodeling characterized by myosin heavy chain isoform switch and fibrosis, LV functional impairment, and decreased dobutamine contractile reserve.^30^ These observations are consistent with, and lend mechanistic support to, our clinical findings.^30^

Air pollution disproportionately affects vulnerable populations (eg, the elderly and those with preexisting cardiorespiratory disease) and differences in underlying vulnerability may affect the risk of developing a health effect from pollution exposure.^31^ Strikingly, we found that women seem to be more vulnerable to the adverse cardiac remodeling effects of air pollutant exposure. We also found that air pollution exposure was associated with elevated LV mass and reduced LV ejection fraction, which are changes that would also be consistent with the effects of hypertension. Patients with the highest exposure to NO_2_ were more likely to have a history of hypertension. However, for the majority of our cohort, blood pressures were in normal range. It is possible therefore that air pollution makes all patients, but particularly women with DCM, more vulnerable to the effects of any elevations in blood pressure, even within seemingly normal ranges and this requires further study.

There are limitations to our study. We were unable to control for potentially important confounders such as diet, activity level, and occupation, all of which are potential risk factors for cardiovascular disease and associated with air pollution exposure. We did, however, control for socioeconomic status, a recognized important potential confounder in air pollution research. However, it is possible that air pollution is the mechanism whereby socioeconomic status is associated with adverse cardiovascular events and studies such as ours contribute to this debate. Although our findings were robust to adjustment for socioeconomic status, smoking status, and geographical clustering by postcode area, without doing a randomized controlled trial, we are unable to eliminate residual confounding completely and our conclusions should be interpreted in this context. Another limitation is index event bias, the event being a DCM diagnosis, where we are looking at prior exposure, which can result in distorted associations. Common to many environmental epidemiological studies, we have estimated ambient air pollution levels based on residential postcode which on average represents 12 households so is a high-resolution measure. However, personal exposure will vary according to individual activity patterns, residential mobility, indoor air pollution levels (including indoor sources of NO_2_ such as cooking), occupational exposure, and exposure during commuting, for which we were unable to account. It is plausible that some of these factors (eg, work patterns, cooking exposure) could contribute to the observed sex differences in adverse cardiac remodeling. We are taking a snapshot of air pollution exposure at 1 time point and assuming that exposure is constant over the follow-up period. We further assume that spatial exposure contrasts are the same over time, which may not be true owing to variability in meteorology and source emissions. Extrapolated exposure values were, however, evaluated previously against measurements of concentrations and showed that this approach was appropriate.^32^ The use of personal sensors to measure air pollution exposure over a long follow-up period could be used to mitigate these limitations, although this would be potentially unrealistic in a cohort of this size. We also focused on NO_2_ and PM_2.5_ initially because these pollutants show the greatest and strongest associations with cardiovascular conditions, and that levels of these pollutants in the UK are close to or exceed World Health Organisation recommended limits. Based on the findings in this study, future studies are planned to explore the major sources and chemical composition of PM_2.5_ on cardiac structure given the high likelihood that different chemical constituents of PM will differentially affect biological responses. We did not evaluate for meteorological variables such as temperature because these are most relevant for short-term outcome studies (such as an acute relationship between a pollutant exposure spike and subsequent hospitalization), which was not the design of this study.

This study was conducted in the UK, where overall pollution levels remain relatively low, compared with other nations such as India or China. Whether this association is seen in regions of high air pollution remains unclear and should be a key focus of future research. Our study was also not powered to evaluate the effect of air pollutant exposure on major cardiovascular clinical events, which have been predominantly based on large ecological studies with limited phenotyping.^33^ Cumulative exposure over time promotes the development of a chronic underlying vulnerable state, which could augment future cardiovascular risk.^34^ In line with this finding, we evaluated the association of chronic pollution with adverse remodeling, which could contribute to downstream adverse cardiovascular outcomes. To evaluate a link between chronic pollution and long-term outcomes would require a study in the setting of very high pollution levels and sufficiently large cohorts given the likely effect size.^34^

In conclusion, this study demonstrates that increased exposure to predominantly road traffic-related air pollutants is associated with an increased in LV mass and a lower LV ejection fraction in patients with HF owing to DCM. This study provides biological plausibility for the established epidemiological links between pollution and HF and supports previous data from animal models. These findings have global implications for management strategies for patients with HF and for guiding public health policy development, offering the promise that tackling air pollution, particularly from vehicles, may lead to improvements in cardiovascular health in patients with HF.

## Declaration of Interests

DJP declares consultancy fees from ApoPharma; research support from Bayer, ApoPharma, and Siemens; speakers fees from ApoPharma and Bayer. JW declares consultancy fees from MyoKardia and research support from MyoKardia. The remaining authors have no conflicts of interest.

## Appendix Supplementary materials

Image, application 1
