# Supplementary material for: Exposure to Elevated Nitrogen Dioxide Concentrations and Cardiac Remodeling in Patients With Dilated Cardiomyopathy
Source: J Card Fail. 2022 Jun;28(6):924–34. doi: 10.1016/j.cardfail.2021.11.023 (PMC9186493; doi:10.1016/j.cardfail.2021.11.023)

**EXPOSURE TO ELEVATED NITROGEN DIOXIDE CONCENTRATIONS AND CARDIAC REMODELLING IN PATIENTS WITH DILATED CARDIOMYOPATHY**

**Supplementary material**

**Methods**

Air Pollution Exposure

To assign long-term air pollution exposure estimates, we used air pollution concentration maps previously developed for epidemiological analyses based on land use regression models for Great Britain. Maps were available for annual average NO_2_ concentrations in 2009 at 200-m resolution^12^ and for PM_2.5_ in 2010 at 100-m resolution.^13^ Both models were previously validated against monitoring data and showed good agreement between modelled and measured air pollution concentrations.^12,13^

We adopted a method^12^ for forward extrapolation of 2009/2010 air pollution exposure to the year of the study cardiovascular magnetic resonance scan (the baseline scan at study enrolment from 2009 to 2015). The method compares the difference in rural background concentrations at concomitant air pollution monitoring sites from the source year (i.e. the year of the model, in this case 2009 or 2010) and the target year (i.e. the year of the scan) for exposure estimation. Absolute differences are then applied to extrapolate (i.e. reduce/increase) modelled concentrations from source years to target year.

We obtained data from the Automatic Urban and Rural Network (AURN) hosted by the Department for Environment, Food & Rural Affairs for PM_2.5_ concentrations from both rural and suburban background stations in 2010 (n=5) and each event year. NO_2_ measurements from rural background stations in 2009 (n=13) and each event year were from the same source. NO_2_ is a more widely monitored pollutant and we could rely on rural background stations only for extrapolation purposes.

Long-term ambient air pollution exposure estimates in the year of scan were assigned to each residential postcode centroid (on average 12 households). We used the participant’s residential postcode at the time of study recruitment. Postcodes were geocoded using year-specific coordinates based on the UK Small Area Health Statistics Unit historical postcode database. Postcode centroids were overlaid with each air pollution surface to obtain NO_2_ and PM_2.5_ estimates for each postcode and concentrations extrapolated to the year of scan using information from the national air pollution monitoring network.

The Table below shows the year-to-year variability of average concentrations obtained from the AURN monitoring stations which were used to for-ward extrapolate from year of air pollution model to year of scan:

|  | **2010** | **2011** | **2012** | **2013** | **2014** | **2015** |
| --- | --- | --- | --- | --- | --- | --- |
| **NO_2_** | 9.37 | 8.49 | 8.51 | 8.13 | 7.51 | 6.89 |
| **PM_2.5_** | 11.17 | 12.28 | 11.28 | 12.86 | 11.79 | 8.25 |

Model Building – LV mass:

To build the baseline model for indexed LV mass (LVMi), biological and clinical variables that could plausibly be associated with LV mass were selected for univariable analysis. These were age, sex, a history of hypertension, race, titin truncating variants, systolic and diastolic blood pressure (as measured on the day of recruitment), and CMR features of left ventricular non-compaction (LVNC).

Variables significant at a p value threshold of inclusion of <0.10 were retained, and then reverse stepwise selection performed with a threshold p value for exclusion of >0.05. Previously discarded variables were added to the final model to assess for changes to the coefficients or significance threshold of retained variables. This resulted in the final model of sex, age, truncating variant in the titin gene, hypertension and socio-economic status. Age and socio-economic status were forced into the model as important confounders (Supplementary Table 4).

Model Building - LVEF:

To build the baseline model for left ventricular ejection fraction (LVEF), biological and clinical variables that could plausibly be associated with LVEF were selected for univariable analysis. These were age, gender, family history of DCM, the presence of left bundle branch block, the presence of mid-wall fibrosis LGE, titin truncating variant, a history of alcohol excess, a history of atrial fibrillation and heart failure medication use (beta blocker, ACE inhibitor and aldosterone antagonist). Medication was included to control for the potential confounding effect of prognostic heart failure medication. Diuretic use or NYHA class were not evaluated as predictors of LVEF as whilst they are associated with LVEF severity, they do not contribute to LVEF impairment. RVEF was not evaluated as it is highly correlated with LVEF (r=0.56, p <0.0001). The presence of mitral regurgitation was also not evaluated as a predictive variable of LVEF because primary valvular disease was an exclusion criteria, meaning that any mitral regurgitation reflected LV dilation and impairment and was not a primary contributor. Any variables with p<0.1 from the univariable analysis were considered for inclusion in an optimised multivariable model, which was created by backward stepwise selection with a threshold p value for exclusion of >0.05 until only significant variables were included the model. Previously discarded variables were added to the final model to assess for changes to the coefficients or significance threshold of retained variables. This resulted in the final model of age, sex, beta blocker use, aldosterone antagonist use and socioeconomic status. Age and socio-economic status were forced into the model as important confounders (Supplementary Table 5).

**Supplementary Table 1:** Cohort demographics and cardiac imaging variables at baseline, stratified by exposure to NO_2_ greater than European legal limits. Data are shown as median [interquartile range] and counts (percentages). BSA, body surface area. NYHA = New York Heart Association functional class.

| Phenotype | NO2 exposure <40ug/m3 | NO2 exposure >40ug/m3 | p |
| --- | --- | --- | --- |
| n | 490 | 169 |  |
| Age at scan (years) (median [IQR]) | 54 [44, 63] | 55 [45, 66] | 0.163 |
| Sex = Male (%) | 331 ( 68) | 104 (62) | 0.184 |
| Ethnicity (%) |  |  | <0.001 |
| Caucasian | 444 ( 91) | 124 (73) |  |
| Weight (kg) (median [IQR]) | 83 [72, 96] | 80 [68, 95] | 0.055 |
| Systolic BP (mmHg) (median [IQR]) | 121 [110, 134] | 124 [110, 137] | 0.662 |
| Diastolic BP (mmHg) (median [IQR]) | 73 [65, 82] | 73 [65, 84] | 0.782 |
| NYHA class (%) | |  | 0.446 |
| 1 | 214 ( 46) | 62 (39) |  |
| 2 | 182 ( 39) | 73 (45) |  |
| 3 | 68 ( 14) | 24 (15) |  |
| 4 | 6 ( 1) | 2 ( 1) |  |
| Controlled hypertension (%) | 131 ( 27) | 61 (36) | 0.027 |
| Diabetes mellitus (%) | 66 ( 13) | 14 ( 8) | 0.1 |
| Beta blocker use (%) | 345 ( 70) | 121 (72) | 0.845 |
| ACE inhibitor use (%) | 388 ( 79) | 139 (82) | 0.455 |
| Aldosterone antagonist use (%) | 174 ( 36) | 66 (39) | 0.464 |
| Diuretic use (%) | 208 ( 42) | 96 (57) | 0.002 |
| Left ventricular ejection fraction (%) | 42 [30, 50] | 37 [28, 47] | 0.007 |
| Left ventricular end diastolic volume indexed to BSA (mL/m^2^) | 117 [103, 141] | 118 [103, 148] | 0.2 |
| Left ventricular end systolic volume indexed to BSA (mL/m^2^) | 67 [52, 96] | 78 [56, 104] | 0.024 |
| Left ventricular stroke volume indexed to BSA (mL/m^2^) | 48 [38, 58] | 47 [36, 54] | 0.111 |
| Left ventricular mass indexed to BSA (g/m^2^) | 85 [73, 105] | 89 [75, 108] | 0.097 |
| Right ventricular ejection fraction (%) | 54 [44, 61] | 54 [41, 62] | 0.826 |
| Right ventricular end diastolic volume indexed to BSA (mL/m^2^) | 84 [70, 101] | 85 [70, 100] | 0.828 |
| Right ventricular end systolic volume indexed to BSA (mL/m^2^) | 40 [29, 52] | 40 [29, 54] | 0.952 |
| Right ventricular stroke volume indexed to BSA (mL/m^2^) | 43 [36, 53] | 43 [34, 51] | 0.467 |
| Mid wall myocardial fibrosis (detected on late gadolinium enhancement imaging) | 166 ( 34) | 61 (36) | 0.668 |
| Left atrial volume indexed to BSA (mL/m^2^) | 56 [46, 70] | 55 [43, 75] | 0.869 |
| Mean septal wall thickness (mm) | 10 [8, 11] | 10 [8, 11] | 0.424 |
| Mean lateral left ventricular wall thickness (mm) | 8 [6, 9] | 8 [7, 9] | 0.125 |
| Mean septal wall thickness (mm) | 5 [4, 6] | 6 [5, 7] | 0.178 |
| Truncating variant in the titin gene (%) | 62 (13) | 18 (11) | 0.582 |
| IMD 2015 score (median [IQR]) | 21 [14, 28] | 16 [9, 23] | <0.001 |
| IMD 2015 quintile (%) |  |  | <0.001 |
| 1 | 23 (5) | 23 (17) |  |
| 2 | 71 (17) | 37 (27) |  |
| 3 | 83 (20) | 24 (18) |  |
| 4 | 111 (26) | 30 (22) |  |
| 5 | 133 (32) | 21 (16) |  |

**Supplementary Table 2:** Univariable linear regression analysis evaluating association between indices of cardiac structure or function and nitrogen dioxide (NO_2_).

| **Phenotype** | **Beta coefficient – phenotype change per interquartile range increase in NO_2_** | **Standard error** | **P value** |
| --- | --- | --- | --- |
| **LVEF (%)** | -1.9 | 0.6 | 0.004 |
| **LVEDVi (mL/m^2^)** | 3.2 | 1.9 | 0.09 |
| **LVESVi (mL/m^2^)** | 4.2 | 1.9 | 0.03 |
| **LVSVi (mL/m^2^)** | -1.0 | 0.7 | 0.14 |
| **LVMi (g/m^2^)** | 4.5 | 1.4 | 0.001 |
| **RVEF (%)** | -0.5 | 0.7 | 0.52 |
| **RVESVi (mL/m^2^)** | -0.6 | 1.1 | 0.63 |
| **RVEDVi (mL/m^2^)** | -1.0 | 1.3 | 0.42 |
| **RVSVi (mL/m^2^)** | -0.6 | 0.7 | 0.41 |

Abbreviations: L/RVEF= left/right ventricular ejection fraction, L/RVEDVi = left/right ventricular end diastolic volume indexed to body surface area, L/RVESVi = left/right ventricular end systolic volume indexed to body surface area, L/RVSVi = left/right ventricular stroke volume indexed to body surface area, LVMi = left ventricular mass indexed to body surface area.

**Supplementary Table 3:** Univariable linear regression analysis evaluating association between indices of cardiac structure or function and particulate matter less than 2.5 µm (PM_2.5_).

| **Phenotype** | **Beta coefficient – phenotype change per interquartile range increase in PM_2.5_** | **Standard error** | **P value** |
| --- | --- | --- | --- |
| **LVEF (%)** | -1.1 | 0.6 | 0.05 |
| **LVEDVi (mL/m^2^)** | 0.8 | 1.7 | 0.65 |
| **LVESVi (mL/m^2^)** | 1.8 | 1.7 | 0.28 |
| **LVSVi (mL/m^2^)** | -1.0 | 0.63 | 0.10 |
| **LVMi (g/m^2^)** | 2.8 | 1.2 | 0.02 |
| **RVEF (%)** | -0.05 | 0.65 | 0.94 |
| **RVESVi (mL/m^2^)** | -1.5 | 1.0 | 0.16 |
| **RVEDVi (mL/m^2^)** | -2.3 | 1.5 | 0.04 |
| **RVSVi (mL/m^2^)** | -1.0 | 0.6 | 0.11 |

Abbreviations: L/RVEF= left/right ventricular ejection fraction, L/RVEDVi = left/right ventricular end diastolic volume indexed to body surface area, L/RVESVi = left/right ventricular end systolic volume indexed to body surface area, L/RVSVi = left/right ventricular stroke volume indexed to body surface area, LVMi = left ventricular mass indexed to body surface area.

**Supplementary Table 4:** Univariable and multivariable linear regression analysis evaluating predictors of left ventricular mass and association between left ventricular mass and nitrogen dioxide (NO_2_). *Adjusted for variables shown in Table. The IQR for NO_2_ concentration was 24.1 to 40.6 μg/m^3^.

|  | **Unadjusted Analysis** | | | **Adjusted Analysis*** | | |
| --- | --- | --- | --- | --- | --- | --- |
|  | **Estimated change in indexed left ventricular mass (g/m^2^)** | **95% Confidence Interval** | **p Value** | **Estimated change in indexed left ventricular mass (g/m^2^)** | **95% Confidence Interval** | **p Value** |
| NO_2_ (per 1 IQR increase) | 4.5 | 1.8 to 7.1 | 0.001 | 4.3 | 1.9 to 7.0 | 0.002 |
| Male | 17.7 | 13.9 to 21.5 | <0.0001 | 17.7 | 13.7 to 21.7 | <0.00001 |
| History of hypertension | 6.8 | 2.7 to 11.0 | 0.001 | 6.1 | 1.7 to 10.5 | 0.006 |
| Age (per 1 year) | 0.07 | -0.06 to 0.2 | 0.25 | 0.007 | -0.14 to 0.15 | 0.92 |
| Truncating variant in titin gene | -7.8 | -13.7 to -1.8 | 0.01 | -8.1 | -14.1 to -2.1 | 0.008 |
| Index of Multiple Deprivation (per quintile) | -0.71 | -2.3 to 0.8 | 0.37 | -0.3 | -1.8 to 1.3 | 0.75 |

**Supplementary Table 5:** Univariable and multivariable linear regression analysis evaluating predictors of left ventricular ejection fraction and association between left ventricular ejection fraction and nitrogen dioxide (NO_2_). *Adjusted for variables shown in Table. The IQR for NO_2_ concentration was 24.1 to 40.6 μg/m^3^.

|  | **Unadjusted Analysis** | | | **Adjusted Analysis*** | | |
| --- | --- | --- | --- | --- | --- | --- |
|  | **Estimated change in left ventricular ejection fraction (%)** | **95% Confidence Interval** | **p Value** | **Estimated change in left ventricular ejection fraction (%)** | **95% Confidence Interval** | **p Value** |
| NO_2_ (per 1 IQR increase) | -1.9 | -3.1 to -0.6 | 0.004 | -1.5 | -2.7 to -0.2 | 0.02 |
| Age (per 1 year) | -0.08 | -0.14 to -0.02 | 0.01 | -0.05 | -0.11 to 0.01 | 0.12 |
| Male | -3.6 | -5.5 to -1.7 | 0.0002 | -3.7 | -5.6 to -1.9 | <0.0001 |
| Beta blocker use | -6.4 | -8.3 to -4.5 | <0.0001 | -4.0 | -6.1 to -2.0 | 0.0001 |
| Aldosterone antagonist use | -7.9 | -9.7 to -6.2 | <0.0001 | -7.0 | -8.9 to -4.9 | <0.0001 |
| Index of Multiple Deprivation (per quintile) | 0.55 | -0.2 to 1.3 | 0.14 | 0.25 | -0.47 to 0.97 | 0.49 |

**Supplementary Table 6:** Univariable and multivariable linear regression analysis evaluating predictors of left ventricular mass and association between left ventricular mass and particulate matter less than 2.5 µm (PM_2.5_). *Adjusted for variables shown in Table. The IQR for PM_2.5_ was 14.3 to 16.3 μg/m3.

|  | **Unadjusted Analysis** | | | **Adjusted Analysis*** | | |
| --- | --- | --- | --- | --- | --- | --- |
|  | **Estimated change in indexed left ventricular mass (g/m^2^)** | **95% Confidence Interval** | **p Value** | **Estimated change in indexed left ventricular mass (g/m^2^)** | **95% Confidence Interval** | **p Value** |
| PM_2.5_ (per 1 IQR increase) | 2.8 | 0.4 to 5.2 | 0.03 | 2.7 | 0.3 to 5.1 | 0.02 |
| Male | 17.7 | 13.9 to 21.5 | <0.0001 | 17.7 | 13.7 to 21.7 | <0.00001 |
| History of hypertension | 6.8 | 2.7 to 11.0 | 0.001 | 6.2 | 1.8 to 10.6 | 0.005 |
| Age (per 1 year) | 0.07 | -0.06 to 0.2 | 0.25 | 0.02 | -0.1 to 0.2 | 0.71 |
| Truncating variant in titin gene | -7.8 | -13.7 to -1.8 | 0.01 | -7.8 | -13.9 to -1.8 | 0.01 |
| Index of Multiple Deprivation (per quintile) | -0.71 | -2.3 to 0.8 | 0.37 | -0.60 | -2.1 to 0.9 | 0.44 |

**Supplementary Table 7:** Mixed effects modelling using postcode area as clustering variable.

| Exposure | Outcome | Variables adjusted for | Intraclass correlation coefficient |
| --- | --- | --- | --- |
| Nitrogen dioxide | Indexed left ventricular mass | Age, sex, hypertension, titin truncating variant, hypertension, socioeconomic status | 0 |
| Nitrogen dioxide | Left ventricular ejection fraction | Age, sex, beta blocker use, aldosterone antagonist use, socioeconomic status | 0.008 |
| Particulate matter diameter <2.5µm | Indexed left ventricular mass | Age, sex, hypertension, titin truncating variant, hypertension, socioeconomic status | 0.001 |

**Supplementary Figure 1:** Univariable association between particulate matter less than 2.5 µm (PM_2.5_) concentrations and cardiac morphology and function. Abbreviations: LV/RV= left/right ventricle. EF= ejection fraction. EDV/ESV= end diastolic/systolic volume. LVM=left ventricular mass. SV= stroke volume. i=indexed to body surface area.


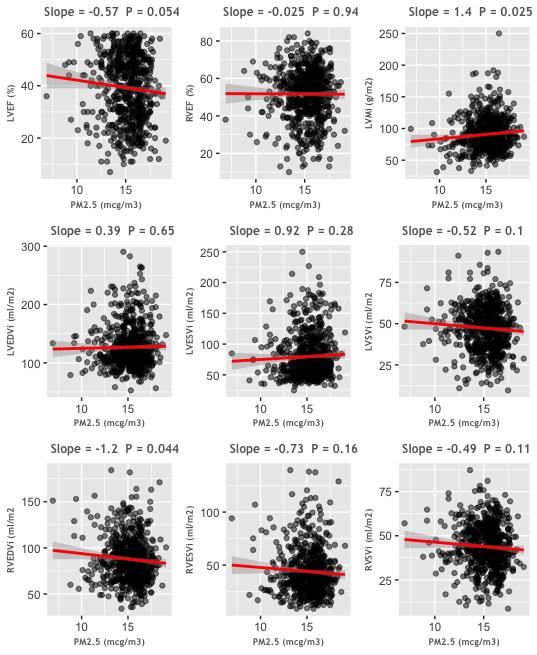


**Supplementary Figure 2:** Univariable association between nitrogen dioxide (NO_2_) concentrations and cardiac morphology and function. There is an association between higher NO_2_ levels and lower left ventricular ejection fraction and higher left ventricular mass. Abbreviations: LV/RV= left/right ventricle. EF= ejection fraction. EDV/ESV= end diastolic/systolic volume. LVM=left ventricular mass. SV= stroke volume. i=indexed to body surface area.


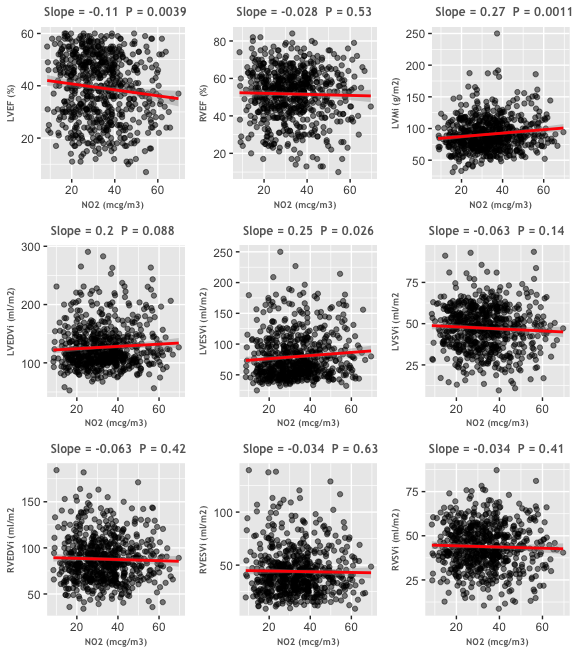


**Supplementary Figure 3:** Cubic splines regression to evaluate alternative dose: response relationships between exposures and outcomes. Restricted cubic spline transformation of exposure variables nitrogen dioxide (NO2) and particulate matter (PM2.5) to investigate non linear relationships with outcomes (indexed left ventricular mass and left ventricular ejection fraction). Plots showing (i) 3 knots and (ii) 5 knots. There are no clear non linear relationships except for where there are few data points at extreme exposure values.


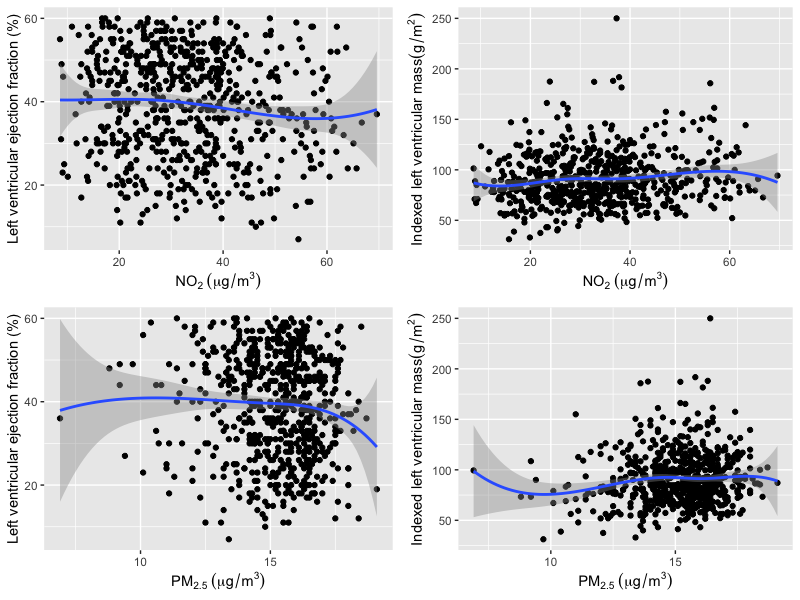


(ii)


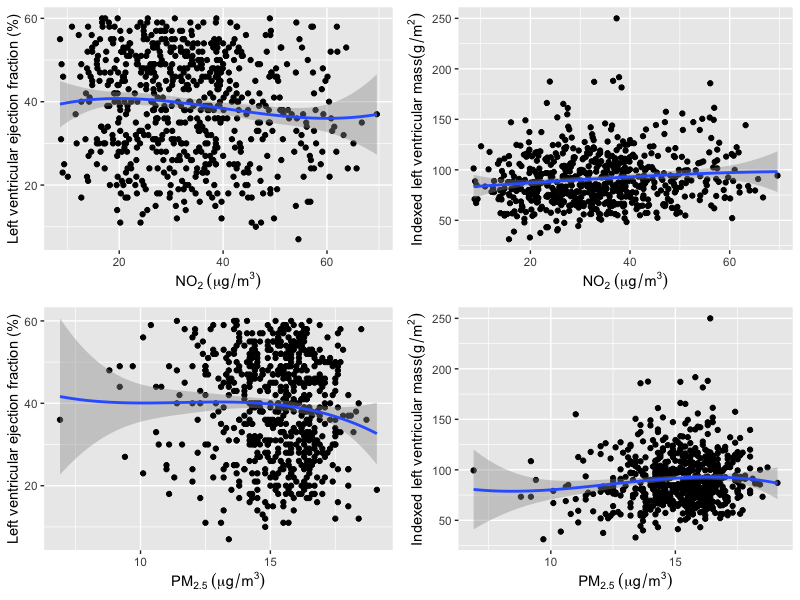

Supplement: Supplementary file 1 [file mmc1.docx]
